# Versatility of 7-Substituted Coumarin Molecules as Antimycobacterial Agents, Neuronal Enzyme Inhibitors and Neuroprotective Agents

**DOI:** 10.3390/molecules22101644

**Published:** 2017-09-30

**Authors:** Erika Kapp, Hanri Visser, Samantha L. Sampson, Sarel F. Malan, Elizabeth M. Streicher, Germaine B. Foka, Digby F. Warner, Sylvester I. Omoruyi, Adaze B. Enogieru, Okobi E. Ekpo, Frank T. Zindo, Jacques Joubert

**Affiliations:** 1 School of Pharmacy, Faculty of Natural Sciences, University of the Western Cape, Cape Town, Bellville 7550, South Africa; ekapp@uwc.ac.za (E.K.); sfmalan@uwc.ac.za (S.F.M.); 3002811@myuwc.ac.za (G.B.F.); frankzindo@gmail.com (F.T.Z.); 2 DST/NRF Centre of Excellence in Biomedical Tuberculosis Research, SA MRC Centre for Tuberculosis Research, Division of Molecular Biology and Human Genetics, Faculty of Medicine and Health Sciences, University of Stellenbosch, Cape Town, Tygerberg 7505, South Africa; hvisser@sun.ac.za (H.V.); ssampson@sun.ac.za (S.L.S.); lizma@sun.ac.za (E.M.S.); 3 Medical Research Council/National Health Laboratory Service/University of Cape Town Molecular Mycobacteriology Research Unit, Department of Science and Technology/National Research Foundation Centre of Excellence for Biomedical Tuberculosis Research, Institute of Infectious Disease and Molecular Medicine and Department of Clinical Laboratory Sciences, University of Cape Town, Cape Town, Rondebosch 7700, South Africa; digby.warner@uct.ac.za; 4 Department of Medical Biosciences, University of the Western Cape, Cape Town, Bellville 7550, South Africa; 3405455@myuwc.ac.za (S.I.O.); 3581698@myuwc.ac.za (A.B.E.); oekpo@uwc.ac.za (O.E.E.)

**Keywords:** coumarin, *Mycobacterium tuberculosis*, cholinesterase inhibitor, monoamine oxidase B inhibitor, structure-activity relationship, albumin binding, neuroprotection

## Abstract

A medium-throughput screen using *Mycobacterium tuberculosis* H37Rv was employed to screen an in-house library of structurally diverse compounds for antimycobacterial activity. In this initial screen, eleven 7-substituted coumarin derivatives with confirmed monoamine oxidase-B and cholinesterase inhibitory activities, demonstrated growth inhibition of more than 50% at 50 µM. This prompted further exploration of all the 7-substituted coumarins in our library. Four compounds showed promising MIC_99_ values of 8.31–29.70 µM and 44.15–57.17 µM on *M. tuberculosis* H37Rv in independent assays using GAST-Fe and 7H9+OADC media, respectively. These compounds were found to bind to albumin, which may explain the variations in MIC between the two assays. Preliminary data showed that they were able to maintain their activity in fluoroquinolone resistant mycobacteria. Structure-activity relationships indicated that structural modification on position 4 and/or 7 of the coumarin scaffold could direct the selectivity towards either the inhibition of neuronal enzymes or the antimycobacterial effect. Moderate cytotoxicities were observed for these compounds and slight selectivity towards mycobacteria was indicated. Further neuroprotective assays showed significant neuroprotection for selected compounds irrespective of their neuronal enzyme inhibitory properties. These coumarin molecules are thus interesting lead compounds that may provide insight into the design of new antimicrobacterial and neuroprotective agents.

## 1. Introduction

Progressive development of resistance to various chemotherapeutic agents used in the management of infectious diseases presents a very serious problem in global public health. Natural products have yielded various classes of effective antimicrobials over the last few decades and continue to play a large role in the identification of novel lead molecules for this purpose [1].

A large variety of coumarin derivatives have been isolated from natural products and an array of pharmacological activities have been described for these compounds, as well as related synthetic coumarins [2,3]. The ability of the coumarin moiety to form several possible interactions with a particular binding site (including hydrogen bonds, electrostatic, π–π and hydrophobic interactions) likely stabilize target binding and contribute to the versatile pharmacological profile of coumarin derivatives. Both the type and placement of functional groups on the coumarin scaffold determine the specific activity of a given derivative [2,3,4].

**Figure 1:**
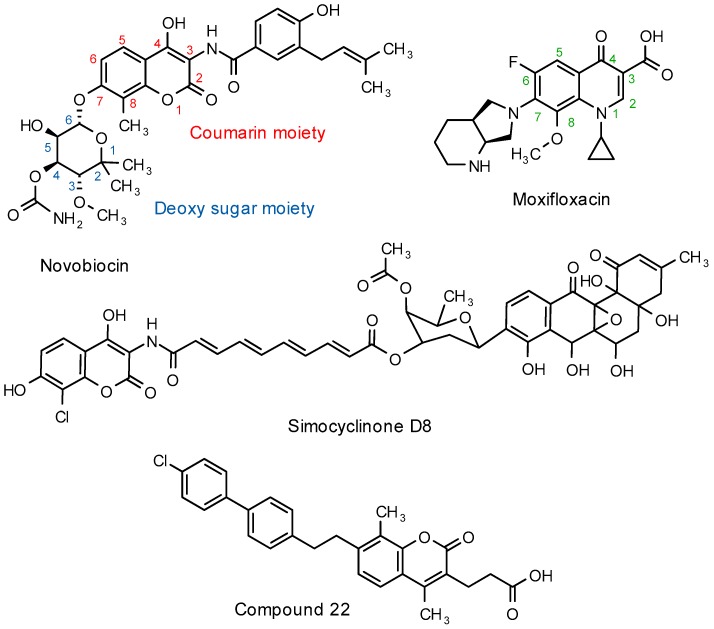
Molecular structure of novobiocin, moxifloxacin, simocyclinone D8 and compound **22** as examples of antimicrobial coumarin derivatives.

Antibacterial and more specifically, antimycobacterial activity are amongst the pharmacological effects that have been described for a range of structurally diverse coumarin derivatives. One prominent example of a coumarin-based antibacterial is novobiocin. Novobiocin, the structurally related chlorobiocin as well as coumermycin A_1_ are 3-amino-4,7-dihydroxycoumarin derivatives, alternatively referred to as the classical aminocoumarin antibiotics. Classical aminocoumarins act as competitive inhibitors of bacterial topoisomerase, more precisely DNA gyrase [5,6]. Competitive inhibition of DNA gyrase by the classical aminocoumarin antibiotics results from an overlap in binding sites between the sugar moiety of the aminocoumarins (Figure 1, numbered in blue) and the adenine ring of adenosine triphosphate (ATP )which is required for the gyrase activity [7]. The non-classical aminocoumarin, simocyclinone D8 (Figure 1), lacks the 7-aminosugar moiety and inhibits bacterial DNA gyrase through simultaneous binding to two alternate binding pockets via interactions with the coumarin moiety and a terminal angucyclinone group. Simocyclinone D8 thus has a unique mechanism of action, despite structural similarities to the standard aminocoumarins [8,9,10]. Similarly, differences in the binding sites between the fluoroquinolone antibiotics (e.g., moxifloxacin which primarily inhibits topoisomerase IV but also interacts with gyrase) allow fluoroquinolone antibiotics to maintain potency in aminocoumarin resistant bacteria and vice versa [5,6,11]. Mycobacterial gyrase, which is a validated drug target in *Mycobacterium tuberculosis*, is also the likely target for the fluoroquinolone class antibiotics as *M. tuberculosis* does not express the topoisomerase IV enzyme [12].

Apart from an interaction with gyrase, bacterial DNA helicase is another suggested target of selected coumarin derivatives [13,14,15]. Like other non-classical coumarin antibiotics, the 7-position on these coumarin derivatives does not contain an amino sugar, but rather a moiety able to undergo hydrophobic interactions with the target site [13,14,15]. Compound **22** (Figure 1), a 4,8–dimethyl-3-propionic acid coumarin derivative with a 2-(4-chloro[1,1-biphenyl]4-yl)ethyl substitution on the 7-position was the most active helicase inhibitor in this series of 7-substituted biphenyl coumarin derivatives [14]. In this series, the methyl substitution in position 4 of the coumarin structure drastically increased the anti-helicase activity of the compounds. 

**Figure 2:**
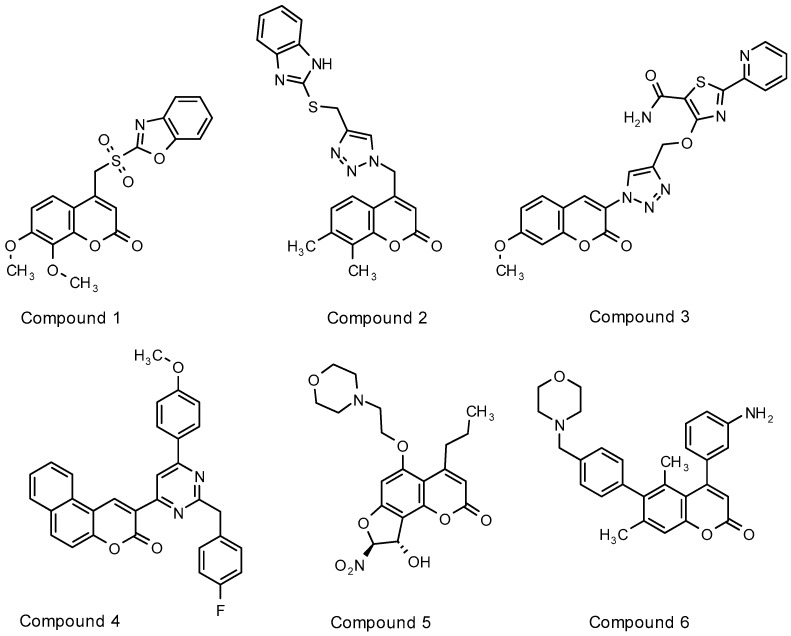
Selected coumarin derivatives demonstrating promising *Mycobacterium tuberculosis* activity across various assays.

Various reports have been published on coumarin derivatives with antimycobacterial activity. Although most studies adequately describe and quantify the activity for respective series of coumarin derivatives, differences in assay methods prevent a direct comparison of antimycobacterial activity of these molecules. Novobiocin as discussed above, demonstrated a minimum inhibitory concentration (MIC) of approximately 6.5 µM in the standard laboratory strain of *M. tuberculosis*, H37Rv [16]. Figure 2 depicts some of the most active compounds in their particular series as evaluated in *M. tuberculosis*. Compound **3** [17] was identified as a likely mycobacterial gyrase inhibitor whereas inhibition of FadD32, an enzyme involved in mycolic acid biosynthesis, was confirmed as target for compound **6** [4]. Mechanisms of action were not suggested for compounds **1** [18], **2** [19], **4** [20] and **5** [21].

As can be expected for compounds which do not necessarily attain their antimycobacterial effect through interaction with the same target sites, structural features important for activity differ between the respective series of compounds. Ultimately, various types and combinations of substitutions on all but positions 1 and 2 of the coumarin scaffold yielded generally effective antimycobacterials, though possibly through different mechanisms of action. This versatile nature of the coumarin scaffold may promote interaction with unique, or possibly multiple targets within the mycobacterial bacilli. 

Unfortunately, structure-activity relationships for the activity of various classes of coumarin derivatives (e.g., central nervous system acting, anticoagulant, and anti-cancer agents) often overlap with that for potent antimicrobial activity [3,22]. A number of review papers describe the importance of coumarin compounds in the field of neurodegenerative disorders where they have shown inhibitory properties towards monoamine oxidases, cholinesterases, β- and *γ*-secretase, and other targets involved in the progression of neurodegenerative disorders [23,24,25]. Similarly, coumarin derivatives described in this article also demonstrated neuronal enzyme inhibitory properties [26]. Therefore, the effective utilization of the coumarin scaffold may essentially be hampered by its versatility unless sufficient selectivity can be ensured. Early identification and structure-activity relationship evaluation of alternative pharmacological effects, over-and-above standard cytotoxicity assays, may increase the viability of coumarin-based antimycobacterial and/or neuroprotective agents.

As part of a collaborative project, a number of 7-substituted coumarin derivatives initially designed and synthesized as multifunctional neuronal enzyme inhibitors [26] were found to exhibit promising antimycobacterial activity (see Table 1) The compounds, originally designed to incorporate coumarin structures known to show monoamine oxidase B (MAO-B) inhibition [27] and selected structural elements of donepezil, a selective acetylcholinesterase (AChE) inhibitor [28], were able to selectively inhibit human MAO-B as well as electric eel AChE and equine butyrylcholinesterase (BuChE) [26]. Further evaluation of the structure-activity relationships for selectivity and specific characteristics of these compounds were therefore required in order to assess the viability of the compounds as neuronal enzyme inhibitors and/or antimycobacterial clinical agents. This article describes the antimycobacterial activity, plasma protein binding properties, cytotoxicity and neuroprotection data for the series of neuronal enzyme inhibitors. Preliminary evaluations on antimycobacterial activity in drug resistant *M. tuberculosis* are described and structure-activity relationships for neuronal enzyme inhibition versus antimycobacterial activity as well as a paired analysis of the neuroprotective properties of selected derivatives are discussed. 

## 2. Results and Discussion

### 2.1. Medium Throughput Screen

A medium throughput screen (MTS) of various compounds from the University of the Western Cape School of Pharmacy drug-design compound library was done to identify novel antimycobacterial agents. A large proportion of the compounds which demonstrated potential antimycobacterial activity in the initial MTS, were coumarin derivatives. A total of 11 coumarin derivatives inhibited more than 50% mycobacterial growth at 50 µM. Table 1 provides the structures and activity of two series of coumarin derivatives **CM1**–**CM19** identified in the MTS, as classified based on the substitution on the 7-position of the coumarin scaffold. The compound numbering from our library was maintained and the derivatives sorted in order of potency within their respective series. Antimycobacterial activity is indicated as the percentage mycobacterial growth on day 5 at 100 µM, 50 µM and 1 µM of the respective derivatives. 

Based on this screen, series 1 seems to contain more potent inhibitors of *M. tuberculosis* with the *p*-bromo-*N*-benzylpiperizine derivatives demonstrating higher activity than the benzyl substituted piperidine derivatives. The exception to this is **CM13** with a chloro substitution on the R^3^ position which reduces the antimycobacterial activity of the compound in this whole cell assay. Interestingly the chloro substitution on R^3^ improves the activity in the piperidine type compound, though it should be noted that the simultaneous removal of the bromine substitution may have contributed to the change in activity. The small activity differences between the 6 most active compounds in series 1 do not allow for prediction of structure-activity relationships within this range. It would however seem that the *p*-bromo-*N*-benzylpiperizine derivatives can tolerate various large and small substitutions in position 4 of the coumarin scaffold relatively well. No significant differences in activity were observed between the trifluoromethane, methyl or unsubstituted 4-substituted coumarin derivatives for this series. This is not the case with series 2, where the trifluoromethyl group in position 4 of the coumarin scaffold seems to decrease the activity when compared to a methyl substitution in this position. The most active compound in series 2 is the R^2^-methyl substituted benzyl-ether (**CM17**) followed by a similar bromobenzyl-ether (**CM2**). Replacement of the hydrogen in position 3 of the coumarin scaffold with a nitrile moiety does not seem to influence activity, but a chloro-substitution in this position decreases activity similar to what was observed for the *p*-bromo-*N*-benzylpiperizine derivatives in series 1. The combination of halogens in R^1^ and R^3^ with a methyl substitution on R^2^ seems to decrease activity in both series 1 and 2.

Comparison of the compounds evaluated in this study with other antimycobacterial coumarin derivatives described in literature (see Figure 2, Section 1) highlighted minor similarities and trends. Many of the more active derivatives described in literature only have small groups substituted at positions 5, 6, 7 and 8 of the coumarin scaffold whereas the compounds evaluated in this study carry the larger substitution at 7 position. Bisubstitution on these positions seems to increase activity. Compound **1** [18], a 4-substituted sulfonyl-benzoxazole derivative was more active when substituted with methoxy groups in both the 7 and 8 positions of the coumarin, than single- or unsubstituted derivatives. Compound **2** [19], a 4-substituted, triazole-linked benzimidazole coumarin with methyl groups at the 5, 7 or 7, 8 positions on the coumarin scaffold similarly demonstrated the highest activity in its series. A common trend for most of these derivatives seems to be a large functional group on position 3 or 4 of the coumarin moiety. Compound **3** [17], bearing a triazole ring on the 3 position of the coumarin scaffold, was able to achieve an MIC value of 6.5 µM. Compound **4** [20], also with a large group on position 3, was the most active in a series of 3-substituted benzocoumarin-pyrimidine hybrid molecules. Series 1 derivatives evaluated in this study likewise seem to tolerate large substitutions on position 3 with no significant differences in activity between substituted or unsubstituted derivatives. In addition to substitutions on position 4, compounds **5** and **6** are substituted with larger morpholine-containing moieties in positions 5 and 6 respectively. Compound **6** [4], bearing a comparatively larger *N*-benzylmorpholine substitution on the 6-position of the coumarin scaffold, was previously shown to inhibit mycobacteria through inhibition of FadD32, a newly validated and promising mycobacterial drug target involved in mycolic acid biosynthesis [29]. Methyl substitutions on positions 5 and 7 were again crucial for activity. The importance of the methyl moieties may be their influence on the geometrical conformation of the adjoining aryl groups, indicating conformation-specific target binding [4]. Similar to the piperidine and piperizine derivatives in series 1 evaluated in this study, compounds **5** and **6** with the larger substitutions on the benzene ring of the coumarin scaffold were substituted with functional groups able to form hydrogen bonds with the target site.

The neuronal enzyme structure-activity relationships of the coumarin derivatives in Table 1 were discussed in detail in our recently published paper [26]. Briefly, the coumarin derivatives substituted at the 7-position with a *N*-benzylpiperidine or *p*-bromo-*N*-benzylpiperazine (series 1) showed better multifunctional neuronal inhibitory activities compared to their 7-benzyl only substituted counterparts (series 2). In addition, the substitution of a nitrile group at position 3 of the coumarin scaffold (**CM9** and **CM14**) improved the multifunctional neuronal inhibitory activities of the compounds in series 1 and the MAO-B activity (**CM4** and **CM19**) in series 2. In both series 1 and 2 substitution at position 4 with a large trifluoromethyl group drastically decreased their neuronal enzymatic activities. It was also noted that, in general, the *N*-benzylpiperidine substituted coumarin derivatives showed significantly improved activities over their *p*-bromo-*N*-benzylpiperazine substituted counterparts. From these studies compound **CM9** was identified as the most promising multifunctional neuronal enzyme inhibitor. It may therefore be possible to increase the selectivity of coumarin derivatives towards either neuronal enzyme inhibitory activity or antimycobacterial property through selective substitution in positions 4 and 7 of the coumarin scaffold.

**Figure 3:**
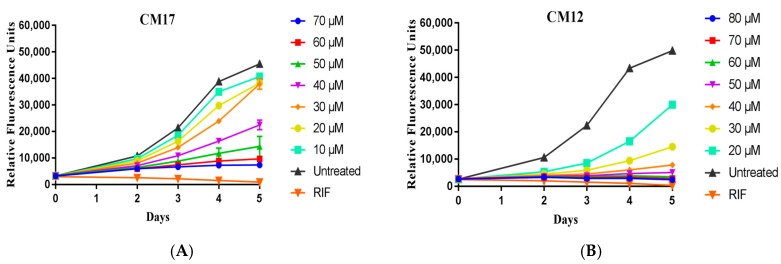
Mycobacterial growth inhibition by compounds **CM12** (**A**) and **CM17** (**B**) at narrower concentration intervals. *M. tuberculosis* H37Rv:pCHERRY3 was cultured in 96 well plates as described, with compounds at the concentrations as shown. Experiments were repeated in biological triplicates; each plot shown here shows a representative biological replicate with the mean and standard deviation of 3 technical replicates for each data point.

All derivatives in series 1 and 2 that demonstrated more than 50% mycobacterial growth inhibition at 50 µM were further analysed at narrower concentration ranges using a similar method to the original MTS. Figure 3 depicts the mycobacterial growth inhibition for compounds **CM12** and **CM17**, which were the most active in their respective series in the initial screen. Relative fluorescence of the far-red fluorescent reporter mCHERRY was used as the indicator of growth during this screen which was performed once to validate the activity found during the initial screen. These graphs show the inhibitory effect of the compounds compared to the untreated as well as a known antimycobacterial rifampicin (RIF) at 6 µM. From this activity validation screen **CM8**, **CM12**, **CM14** and **CM15** (all in series 1) were identified as the most potent compounds and evaluated in further studies to gain insight into their antimicrobacterial properties.

### 2.2. Evaluation of Compound Activity in Quinolone Resistant Mycobacterium tuberculosis

**Figure 4:**
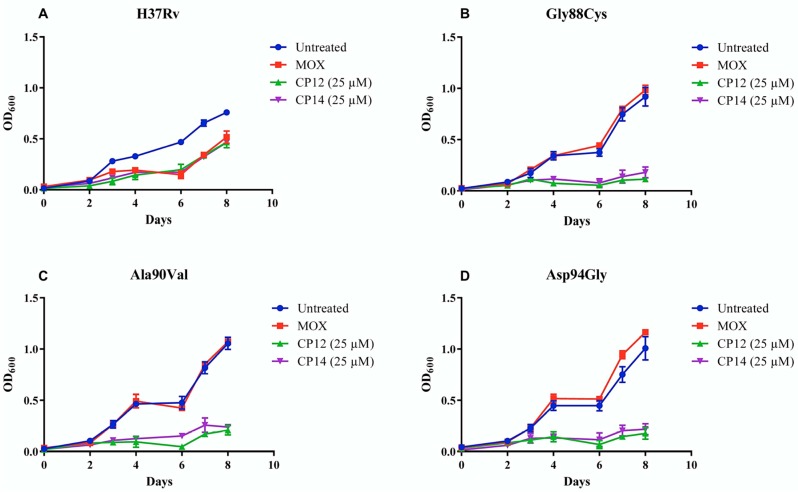
Preliminary evidence of maintained activity of **CM12** and **CM14** in fluoroquinolone sensitive (**A**: H37Rv) and resistant *M. tuberculosis* (**B**, **C**, and **D**: Gly88Cys, Ala90Val, and Asp94Gly, respectively). Moxifloxacin (MOX) was used at a concentration of 1.2 µM. OD_600_: optical density measured at 600 nm.

Various coumarin-based antimicrobials have been shown to target bacterial DNA gyrase, which is also the suggested target of the fluoroquinolone antibiotics in mycobacteria [5,6,11,12]. It was therefore decided to evaluate whether the coumarin derivatives evaluated in this study would be able to maintain potency in fluoroquinolone resistant mycobacteria. The activity of **CM12** and **CM14** were evaluated in three strains (Gly88Cys, Ala90Val and Asp94Gly) of *M. tuberculosis* demonstrating moxifloxacin resistance. Genetic mutations in quinolone-resistance determining regions (QRDR) of DNA gyrase are primarily responsible for conferring resistance to various fluoroquinolone antibiotics. Particularly substitutions in the 94 position are commonly identified in quinolone resistant strains [30]. **CM12** and **CM14** maintained potency in all evaluated strains featuring three different mutations in the QRDR region (see Figure 4). Preliminary investigations indicate that it is likely that compounds in series 1 will maintain activity in fluoroquinolone resistant mycobacteria. Additional evaluations are required to determine the true extent of activity in resistant strains for series 1 as well as series 2 derivatives.

### 2.3. Minimum Inhibitory Concentration Determination

MICs of the most active compounds from the initial evaluations (**CM8**, **CM12**, **CM14**, and **CM15**) were determined independently at the University of Stellenbosch (US, South Africa) and University of Cape Town (UCT, South Africa) utilizing different assay methods described in detail in the methodology section. Briefly, the US assay (similar to the assay used for the MTS), utilized a H37Rv strain transformed with a plasmid encoding a red fluorescent reporter (pCHERRY3 [31]), a gift from Tanya Parish (Addgene plasmid # 24659)), in albumin-containing media (Middlebrook 7H9 broth, Becton, Dickinson and Co., Franklin Lakes, NJ, USA, enriched with 10% oleic albumin dextrose catalase (OADC), 0.2% glycerol and 0.05% Tween 80; 7H9+OADC). The UCT method utilized a standard broth microdilution method with *M. tuberculosis* H37RvMA pMSP12:GFP (green fluorescent protein) in glycerol-alanine-salts with 0.05% Tween 80 and iron (GAST-Fe) [32]. Consistently lower MICs were observed in the GFP GAST-Fe assay. **CM8**, **CM12**, and **CM14** all showed approximately 2- to 3-fold lower MIC_90_ values in the UCT laboratory assay with **CM15** showing the largest discrepancy with a 14-fold difference between the two methods (Table 2). For the purposes of comparison with standard antimycobacterials under similar conditions, **CM12** and **CM14** at 25 µM were able to achieve growth inhibition in similar ranges to moxifloxacin at 1.2 µM whereas RIF at 6 µM resulted in 100% inhibition of bacterial growth in the pCHERRY3 assay. RIF has a MIC of 0.0274 µM in the GFP assay.

Although it is relatively common to see differences in MIC between different media types, the drastic differences, in particularly for compound **CM15**, warranted further investigation. In the assay methods used in this study, differences in the observed MIC could be due to the lack and presence of albumin in GAST-Fe and 7H9+OADC media respectively, the unique reporters used in the two assays, the difference in inoculum size or alternatively, result directly from the mechanism of action of the compounds. The size of the inoculum has been shown to influence the MIC of antimycobacterials. A 10-fold increase in inoculum size resulted in an approximately 4-fold increase in MIC of bedaquiline [33]. The optical density measured at 600 nm (OD_600_) of 0.004 and 0.04 for the GAST-Fe and 7H9+OADC methods respectively, could therefore contribute to the higher MIC observed using the second method.

Another likely contributor to the observed differences in MIC for the coumarin-based derivatives may be binding to albumin. Various coumarin derivatives (e.g., warfarin) are known to bind extensively to blood-soluble proteins. Serum albumins, which are the major soluble protein constituents of the circulatory system (4% *w*/*v*), have the ability to reversibly bind a large variety of exogenous compounds including fatty acids, amino acids, drugs and pharmaceuticals [34,35,36]. Plasma protein binding forms an integral part of distribution and bioavailability for numerous medications currently on the market but might also become problematic where binding is extensive [37,38]. In vitro, binding to albumin would reduce the amount of compound available to exert an effect on the mycobacterial cell and in theory reduce the observed MIC of the derivative. The impact of the albumin binding on in vivo MIC of compounds would however depend on the extent and nature of the binding to albumin within the host. 

### 2.4. Albumin Binding Assay

**Figure 5:**
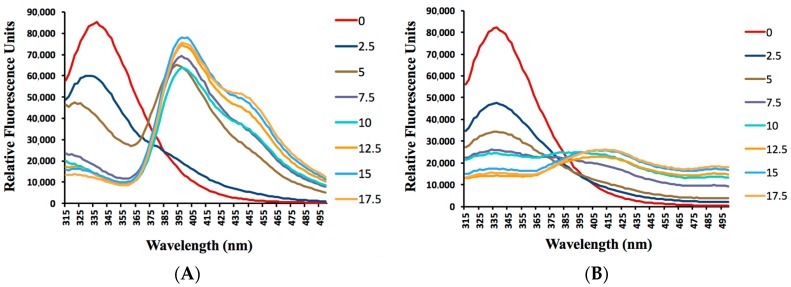
The effects of **CM14** (**A**) and **CM15** (**B**) on the fluorescence spectra of BSA at 22 °C. ex = 295 nm; c(BSA) = 1.00 × 10^−5^ mol L^−1^; c(Q)/(×10^−6^ mol L^−1^). Final concentrations of **CM14** and **CM15** = 0 × 10^−6^ mol L^−1^, 2.5 × 10^−6^ mol L^−1^, 5.0 × 10^−6^ mol L^−1^, 7.5 × 10^−6^ mol L^−1^, 10 × 10^−6^ mol L^−1^, 12.5 × 10^−6^ mol L^−1^, 15.0 × 10^−6^ mol L^−1^, and 17.5 × 10^−6^ mol L^−1^.

To better understand the observed differences in MIC between the two assays, we investigated possible albumin binding for the two coumarin derivatives **CM14** and **CM15** with the largest differences in MIC between the GAST-Fe and 7H9+OADC media based assays. Albumin-enriched media contain approximately 0.5% *w*/*v* bovine serum albumin (BSA). Results of previous studies indicate that human serum albumin (HSA) and BSA are similar proteins in space structure and chemical composition [39,40,41]. BSA was therefore selected as a protein model to investigate fluorescence quenching as an indicator of the extent of interaction between coumarin derivatives **CM14** and **CM15** and albumin, using fluorescence spectroscopy. Fluorescence quenching is the decrease of the quantum yield of fluorescence from a fluorophore induced by a variety of molecular interactions with the quencher molecule. Fluorescence quenching can be dynamic, resulting from collisional encounters between the fluorophore and quencher, or static, resulting from the formation of a ground state complex between the fluorophore and quencher [42,43,44]. The effects of compounds **CM14** and **CM15** on the fluorescence quenching of BSA, excited at 295 nm are presented in Figure 5.

The emission spectrum of **CM14** showed two maxima, the first one at 335 nm (which is characteristic for tryptophan in BSA [45]) and the second peak at 400 nm (which is assigned to fluorescence of compound **CM14** at concentrations of 5 × 10^−6^ mol L^−1^ and upwards). The emission spectrum of **CM15** only showed a maximum at 335 nm for BSA and a weak emission peak of **CM15** at 415 nm at higher concentrations of the compound. The excitation and emission maxima of **CM14** and **CM15** are shown in Table 3.

**Figure 6:**
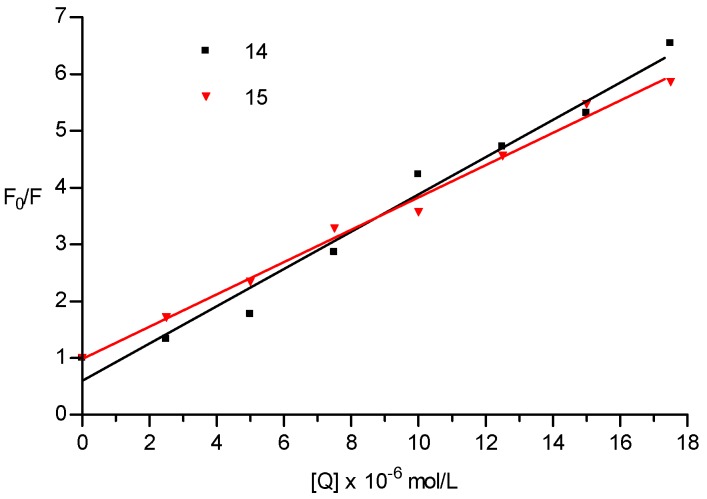
Stern-Volmer plots of BSA (1.00 × 10^−5^ mol L^−1^) quenched by **CM14** and **CM15** at 22 °C.

The mechanism of the fluorescent quenching of **CM14** and **CM15** to BSA is described using the Stern-Volmer equation:

F_0_/F = 1 + k_sv_[Q] = 1 + k_q_ τ_0_[Q]

where F_0_ and F are the fluorescence intensities before and after the addition of the quencher, respectively; k_sv_ is the dynamic quenching constant; k_q_ is the quenching rate constant; [Q] is the concentration of quencher; τ_0_ is the average lifetime of the molecule without quencher and its value is considered to be 10^−8^ s [46,47]. The Stern-Volmer plot of the **CM14** and **CM15** is presented in Figure 6. The plots show a good linear relationship within the investigated concentrations of **CM14** and **CM15**.

The results in Table 3 show that k_q_ values were much greater than the limiting diffusion rate constant of the biomolecule (2.0 × 10^10^ mol L^−1^ S^−1^), which indicated that the probable quenching mechanism of BSA-**CM14** and BSA-**CM15** interactions was initiated by complex formation rather than by dynamic collision [48].

It is evident from the results that the fluorescence intensity of BSA decreases with increasing concentrations of compounds **CM14** and **CM15**. It can also be concluded that the fluorescence quenching of tryptophan in BSA as observed for compounds **CM14** and **CM15** results from complex formation between the two compounds and BSA. The more extensive decrease in fluorescence observed at lower concentrations of compound **CM15** likely indicate a higher binding affinity between compound **CM15** and albumin. These results indicate that albumin binding likely plays a large role in the observed differences in activity between the GAST-Fe and 7H9+OADC assays used in this study. The higher albumin binding affinity of **CM15** is likely the reason for the larger MIC variation perceived for this compound.

In addition to providing more clarity about the observed MICs, albumin binding properties may also provide some insight into the possible pharmacokinetic behavior of these drugs. Binding and dissociation of compounds from plasma proteins is a dynamic process with only the unbound fraction of the drug available to exert an effect. In a clinical setting, the dosage of a drug is calculated to ensure that, at any point in time, sufficient free drug is available to have the required pharmacological effect. The same principle would apply to side effects or toxicity and thus, plasma protein binding becomes an important consideration, particularly in highly bound drugs. In light of the divergent albumin binding affinities observed in the evaluated compounds, it would likely be more pertinent to utilize the MIC values obtained in the albumin free GFP-GAST-Fe media for the purposes of determining structure-activity relationships for antimycobacterial activity. **CM15**, which seems to be at least 3-fold more active than the other top compounds in the GFP assay, has a large CF_3_ substitution on the 4 position of the coumarin scaffold, and a *p*-bromo-*N*-benzylpiperizine substitution on C7. Activity differences between **CM8**, **CM12** and **CM14** are not pronounced enough to draw structure-activity relationship conclusions.

### 2.5. Cell Viability Assays 

Cytotoxicity assays for the compounds showing the lowest MIC_99_ (**CM8**, **CM12**, **CM14**, and **CM15**) were done on Chinese hamster ovary (CHO) epithelial cells using a standard MTT assay method (Table 4) [49]. Data was supplemented with further cytotoxicity analysis of **CM14** and **CM15** as well as **CM9** in a similar assay using the neuronal type SH-SY5Y neuroblastoma cells. The SH-SY5Y cytotoxicity assay results were used to determine the concentration at which the compounds do not affect the viability of the SH-SY5Y cells in order to determine their neuroprotective effect using the 1-methyl-4-phenyl pyridinium (MPP^+^) induced neurotoxicity method [50,51].

**CM9** was included in the neuronal cell viability and MPP^+^ induced neurotoxicity assays because it was identified previously as the best multifunctional neuronal enzyme inhibitor (50% inhibitory concentration (IC_50_): MAO-B = 0.30 µM; AChE = 9.10 µM; BuChE = 5.90 µM) within the series of coumarin derivatives (Table 4) [23]. **CM9** did not show any significant antimycobacterial activity and was therefore not included in the CHO cytotoxicity assay. **CM14** was included to draw a comparison between the neurotoxicity and potential neuroprotective effect of the 3-nitro-4-methylcoumarin derivatives substituted at the 7-position with either a *N*-benzylpiperidine (**CM9**) or *p*-bromo-*N*-piperazine moiety (**CM14**). **CM15**, with a large trifluoromethyl substituent on position 4, showed the most promising antimycobacterial activity with minimal multifunctional neuronal enzyme inhibitory activity. It was included in the neurotoxicity assays to study the effect of the neuronal enzyme inhibitory activities, or lack thereof, on the neuroprotective ability of these compounds or if other mechanisms of action are involved. **CM8** and **CM12** were not included in the neurotoxicity assays as their multifunctional neuronal enzyme inhibitory properties were not as pronounced as **CM9** and **CM14**.

Cytotoxicity (CC_50_, 50% cytotoxic concentration) observed for **CM8**, **CM12**, **CM14** and **CM15** on CHO cells was moderate (CC_50_: 15.5–41.2 µM) indicating low toxicity compared to the cytotoxic agent emetine (CC_50_ = 0.06 µM). These compounds however showed low selectivity indices when both the GFP and mCHERRY MIC_99_ assay results are compared to the CC_50_ results (Table 4). The compounds, in general, were slightly more selective towards the mycobacterial strain when using the GFP assay results, with **CM15** showing the best selectivity (selectivity index (SI) = 3.16). The slight selectivity was however not retained when the results from the mCHERRY assay were used in the SI calculations. These poor selectivity indices may limit the further development of these coumarin derivatives. However, the structure-activity relationships identified in this paper may enable the design of coumarin structures with improved antimycobacterial activities and subsequently more favourable selectivity indices.

**Figure 7:**
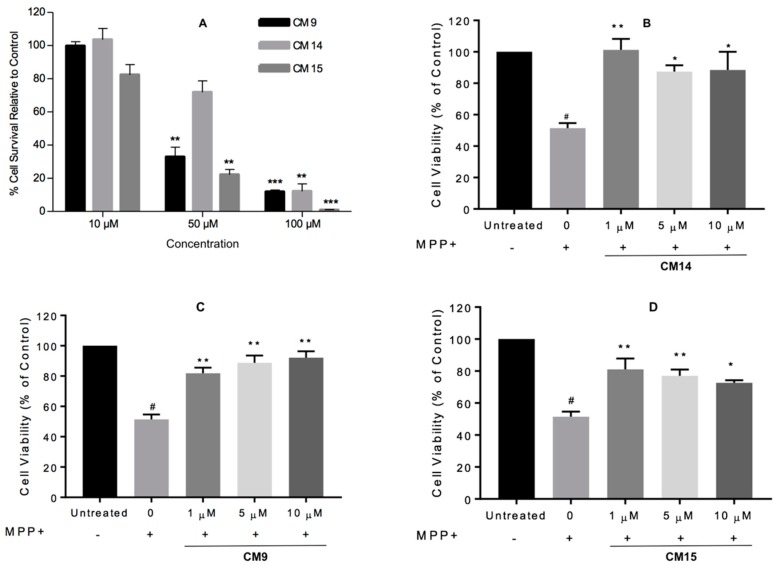
(**A**) Cellular viability of SH-SY5Y cells treated with compounds **CM9**, **CM14** or **CM15** at 10 µM, 50 µM and 100 µM for 48 h. Data is presented as mean ± standard error of mean (SEM). Significance is set at a level of * *p* < 0.05 compared to vehicle treated control cells (taken as 100% cell viability). (**B**–**D**) The effects of compounds **CM9**, **CM14** or **CM15** on MPP^+^-induced (1000 μM) cytotoxicity in SH-SY5Y cells. The viability of the untreated control was defined as 100%. MPP^+^ without test compound showed a significant decrease in cell viability relative to the control (# *p* < 0.05). The level of significance for the test compounds is set at * *p* < 0.05 compared to the MPP^+^ only treated control (0). Statistical analysis (Turkey post hoc test) was performed on all raw data, with asterisks signifying significant inhibitory effect. * *p* < 0.05; ** *p* < 0.001; *** *p* < 0.0001.

The viability of SHSY-5Y human neuroblastoma cells was assessed at different concentrations of compounds **CM9**, **CM14** or **CM15** (Figure 7A). Treatment with the compounds at 10 µM did not significantly affect the viability of the SH-SY5Y cells (*p* > 0.05). However, at higher concentrations (50 µM and 100 µM) the viability of the cells were significantly (*p* < 0.001) affected by the test compounds, although to a lesser extent by **CM14**. These results also correlate to the CC_50_ CHO values obtained for **CM14** and **CM15** (Table 4). 

Based on the SH-SY5Y cytotoxicity analysis it was decided that the MPP^+^ neuroprotection studies would be done at compound concentrations between 1 µM and 10 µM in order maintain the viability of the cells. MPP^+^ is highly toxic to neurons and has been widely used to induce neurodegeneration in various in vitro and in vivo models [50,51]. Several signaling pathways have been suggested to be responsible for MPP^+^-mediated neurotoxicity in SH-SY5Y cells, for instance, trigger of oxidative stress [52], induction of apoptosis [53], and inactivation of pro-survival phosphoinositide 3-kinase (PI3-K)/Akt cascade [54,55,56]. This assay was deemed appropriate to test for initial neuroprotective ability of **CM9**, **CM14** and **CM15** because of the multitude of pathways involved in MPP^+^ mediated neurotoxicity and the multifunctional enzyme inhibitory abilities of the test compounds, especially **CM9**.

SH-SY5Y cells were exposed to the neurotoxin MPP^+^ with or without test compound, and cell viability was evaluated using the MTT mitochondrial function assay [43]. As illustrated in Figure 7B–D, after the exposure to 1000 μM MPP^+^ for 48 h, the cell viability declined significantly to 51.42 ± 3.18%. However, its cytotoxic effects were ameliorated in the presence of **CM9**, **CM14** and **CM15** at 1 µM, 5 µM and 10 µM concentrations. The compounds, at a 1 µM concentration, restored cell survival to 81.86 ± 3.72%, 101.2 ± 6.00% and 81.05 ± 6.83%, respectively. **CM9** showed activity in a dose dependent manner exerting maximal cytoprotection of 92.2 ± 4.18% at 10 µM. At 5 µM and 10 µM a slight decrease in cytoprotection was observed for **CM14** and **CM15**. This may indicate that in the neurotoxin challenged state the SH-SY5Y cells may be more sensitive to the cytotoxic effect of the test compounds. Therefore, **CM14** and **CM15** might have enhanced the toxicity of the neurotoxin which resulted in lower neuroprotection values observed at higher concentrations. Thus, in general and especially at a 1 µM concentration, these compounds, and particularly **CM14**, exerted highly significant cytoprotection towards MPP^+^ insults to the SH-SY5Y neural cells.

The results of the MPP^+^ neuroprotection study did not provide any information on structure-activity relationships of these compounds as **CM9**, **CM14** and **CM15** showed similar levels of cytoprotection. It was however noted that the neuronal enzyme inhibitory activity of these compounds is not the only factor that play a role in cytoprotection and that other mechanisms of action are involved. For instance, **CM15** showed poor neuronal enzyme inhibitory activities but was still able to significantly protect the cells from MPP^+^ induced injury. **CM9** with the best multifunctional enzyme inhibitory profile showed cytoprotection at the same level as **CM15**. As described in the literature coumarin derivatives may possess anti-inflammatory, anti-apoptotic and antioxidant effect [2,3]. These effects combined with the multifunctional neuronal enzyme inhibitory abilities of the compounds may explain the excellent neuroprotection observed. Therefore, further neuroprotection studies are necessary to elucidate the mechanism(s) of action of these compounds.

## 3. Materials and Methods

### 3.1. Compound Synthesis

Synthesis methods of the compounds described in this article are described in detail in previous publications by the drug design group at University of the Western Cape (UWC, South Africa) [26]. 

### 3.2. Initial Medium Throughput In Vitro Activity Screen

This study was approved by the Stellenbosch University Health Research Ethics Committee (S15/06/135). The *M. tuberculosis* H37Rv strain used during this study was obtained from the American Type Culture Collection (ATCC) (ATCC 27294) and whole genome sequencing [57] and virulence confirmation in a murine infection model [58] was used to verify the strain. The episomal plasmid pCHERRY3, a gift from Tanya Parish (Addgene plasmid #24659) [31], which expresses the far-red fluorescent reporter mCHERRY and contains a hygromycin B resistance cassette, was transformed into this electrocompetent *M. tuberculosis* H37Rv. Growth curve analysis of both *M. tuberculosis* H37Rv and *M. tuberculosis* H37Rv:pCHERRY was performed twice to ensure that the plasmid did not have any deleterious effects on growth.

The antimycobacterial activity of the synthetic compounds was determined using a medium throughput 96-well plate assay. *M. tuberculosis* H37Rv with and without the reporter was cultured in 7H9+OADC, without and with 50 µg/mL hygromycin B respectively, whereafter the culture was strained (40 µm; Becton, Dickinson, and Co.), and the OD_600_ was adjusted to 1.0. Culture was added to each well, resulting in a final OD_600_ of 0.02. White, flat bottom 96-well microtiter plates were prepared, with a final volume of 200 µL, final bacterial OD_600_ of 0.02, and final compound concentration of 100 µM, 50 µM or 1 µM. 

Controls included dimethyl sulfoxide (DMSO), positive, negative, and compound control. The background control, which contains *M. tuberculosis* H37Rv without the fluorescent reporter is subtracted from the fluorescence measurements since it displays the inherent fluorescence of *M. tuberculosis* H37Rv. Negative control is included as an untreated fluorescence control where the DMSO control is included to ensure that the maximum amount of DMSO present in the compound dilutions does not interfere with growth. The positive control wells contained 6 µM RIF and the compound controls contained 100 µM compound which was used to confirm that the compound does not autofluoresce in the absence of *M. tuberculosis*. 

One-hundred microliters of the *M. tuberculosis* H37Rv:pCHERRY3 diluted culture was added to each well except the gain, background and compound control wells. In the gain control wells, 100 µL of the undiluted *M. tuberculosis* H37Rv:pCHERRY3 culture was added and in the background control wells *M. tuberculosis* H37Rv without the fluorescent reporter was added. The plates were sealed with breathable sealing film and incubated at 37 °C for 6 days, taking readings on days 0, 2, 3, 4, 5, and 6 using a BMG Labtech POLARstar Omega plate reader (BMG Labtacch, Offenburg, Germany, excitation: 587 nm, emission: 610 nm). During the readings the breathable seal was removed and an optical sealing film was adhered, whereafter it was removed and replaced by a new breathable sealing film. The average of the background control was subtracted from the fluorescence readings whereafter the data were visualised using GraphPad Prism v7.01 (GraphPad software, La Jolla, CA, USA), by plotting the relative fluorescence units (as a measure of growth) over time. The percentage inhibition was also calculated on day 5, by dividing the treated by the untreated values. Compounds that inhibited more than 50% growth at 50 µM were selected for further study and screened at narrower concentration ranges to more accurately ascertain the extent of the compound’s antimycobacterial activity. The second round of screening was also performed using relative fluorescence as a proxy for growth. The growth curves were set up similarly to the initial screen. Five compounds which showed the highest activity during the second screen were selected to undergo further analysis. Of these five compounds, four were coumarin derivatives.

### 3.3. Activity in Moxifloxacin Resistant M. tuberculosis

Three *M. tuberculosis* moxifloxacin resistant mutants, carrying G88C, A90V, or D94G amino acid changes in gyrase A, were tested for CM12 and CM14 cross-resistance. Moxifloxacin stock solutions were prepared at a concentration of 25 µM in DMSO and stored at −80 °C. The resistant mutants and an *M. tuberculosis* H37Rv susceptible strain were prepared as described in Section 3.2 and subsequently exposed to 1.2 µM moxifloxacin, 25 µM CM12 and 25 µM CM14 in technical triplicates in a black, flat, clear-bottom 96 well plate. Each well containing the respective strain was inoculated to an OD_600_ of 0.02 in a final volume of 200 µL. Readings were taken as described in Section 3.2 over 8 days and OD_600_ was plotted over time using GraphPad Prism v7.01.

### 3.4. Minimum Inhibitory Concentration Determination

MICs were determined for the four most active coumarin derivatives identified in the MTS. **CM8**, **CM12**, **CM14**, and **CM15** were screened using two independent methods as described below.

#### 3.4.1. *M. tuberculosis* pMSP12:GFP in GAST-Fe Media

The first MIC determination was performed at the Institute of Infectious Disease and Molecular Medicine, University of Cape Town (UCT) at a screening facility jointly managed by the MRC/NHLS/UCT Molecular Mycobacteriology Research Unit (A/Prof Digby Warner) and the H3D Drug Discovery and Development Unit (Prof Kelly Chibale). Here, the standard broth microdilution method was performed using *M. tuberculosis* H37RvMA pMSP12:GFP [32]. A 10 mL culture was grown in glycerol–alanine–salts with 0.05% Tween 80 and iron (0.05%; GAST-Fe) pH 6.6, to an OD_600_ of 0.6–0.7. Thereafter the culture was diluted 1:100 in GSAT-Fe. In a 96-well microtiter plate, two-fold serial dilutions of each compound were prepared in GAST-Fe whereafter 50 µL of the diluted culture was added to each serial dilution well resulting in an OD_600_ of 0.004. The plate layout used was modified from a previously described method [59]. Controls included a bacterial growth control (5% DMSO in GAST-Fe) and a positive control (0.15 µM RIF). The microtiter plate was incubated at 37 °C with 5% CO_2_ and humidity, sealed within a secondary container. Using a plate reader (FLUOstar OPTIMA, BMG Labtech GmbH, Ortenberg, Germany; excitation: 485 nm; emission: 520 nm) relative fluorescence was measured on days 7 and 14. The CCD Vault from Collaborative Drug Discovery (Burlingame, CA, USA, www.collaborativedrug.com) was used to archive and analyse the fluorescence data. Analyses included normalisation to the bacterial growth and antimycobacterial growth control as the maximum and minimum growth representations respectively generating a dose-response curve using the Levenberg–Marquardt damped least-squares method. This dose response curve was used to calculate the MIC_90_ and MIC_99_ which is described as the lowest concentration at which the compound inhibits growth by more than 90% and 99%, respectively. 

#### 3.4.2. *M. tuberculosis* H37Rv:pCHERRY3 in 7H9+OADC

The second technique used a different fluorescent reporter (mCHERRY) and media (7H9+OADC). Dose-response curve analysis was carried out on the same compounds as above. Dose response curves were performed by exposing *M. tuberculosis* H37Rv:pCHERRY3 to increasing concentrations of each compound in white flat bottom 96 well plates. *M. tuberculosis* H37Rv with and without mCHERRY was prepared as described in Section 3.2. In a white, flat bottom 96 well microtiter plate two-fold serial dilutions of the compounds were prepared with a 100 µL final volume and final bacterial OD_600_ of 0.04. This method was adapted from a previously described method [59]. The controls included were a background control (*M. tuberculosis* H37Rv in 7H9+OADC), a negative control (*M. tuberculosis* H37Rv:pCHERRY3 in 7H9+OADC) and a positive control (*M. tuberculosis* H37Rv:pCHERRY3 in 7H9+OADC containing 2 µM RIF). The microtiter plate was sealed with a breathable film, placed in a secondary container and incubated at 37 °C for 5 days, after which a relative fluorescence measurement (Excitation: 587 nm; Emission: 610 nm) was taken using a BMG Labtech POLARstar Omega plate reader. To analyse the relative fluorescence data the average of the background control was subtracted from each well, and the subsequent data set was analysed in GraphPad Prism v.7.01. Data were first normalised using built in non-linear dose-response curve analysis. This gave an output of IC_50_ as well as a Hill Slope which was used to determine the IC_90_ and IC_99_ of each compound. IC_90_ and IC_99_ was defined as the concentration where 90% and 99% of growth was inhibited, respectively.

### 3.5. Bovine Serum Albumin Binding Assay

The interaction between coumarin derivatives (**CM14** and **CM15**) and BSA were measured by fluorescence spectroscopy. BSA (≥98%) was obtained from Sigma Aldrich (Kempton Park, South Africa) and dissolved in a Tris–HCl (0.10 mol L^−1^, pH = 7.4) buffer to form the BSA solution with a concentration of 1.00 × 10^−5^ mol L^−1^. A Tris–HCl buffer (0.10 mol L^−1^, pH = 7.4) containing 0.10 mol L^−1^ NaCl was selected to keep the pH value constant and to maintain the ionic strength of the solution. **CM14** and **CM15** stock solution (1.00 × 10^−3^ mol L^−1^) was prepared in MeOH. All other reagents were of analytical grade and double-distilled water was used in the experiments. Fluorescence spectra were recorded on a Synergy Mx fluorescence plate reader (BioTek, Winooski, VT, USA). Black 96 well plates were used in all experiments. The excitation and emission slits were 9 nm. Fluorescence spectra were measured in the range of 315–500 nm at the excitation wavelength of 295 nm. All experiments were done at 22 °C. The assay was conducted using a 200 µL solution containing 1.00 × 10^−5^ mol L^−1^ BSA that was titrated by successive additions of 1.00 × 10^−3^ mol L^−1^ coumarin **CM14** or **CM15** solution. The final concentration of the compounds varied from 0 to 1.75 × 10^−5^ mol L^−1^. Titrations were done manually using a micro-pipette in 2 min intervals. Quenching values remained unchanged for more than 10 min after individual titrations. 

### 3.6. Chinese Hamster Ovary Cell Cytotoxicity Assays

Cytotoxicity was evaluated on CHO cells utilizing a standard 3-[4,5-dimethylthiazol-2-yl]-2,5-diphenyl tetrazolium bromide (MTT) assay [49]. The MTT is a yellow tetrazolium salt that is reduced to purple formazan in the presence of living cells. This reduction reaction was used to measure growth and chemosensitivity. The test samples were tested in triplicate on one occasion. Cells were seeded in microtiter plates at a density of 10^5^ cells/mL and incubated for 24 h prior to exposure. The test samples were prepared to a 20 mg/mL stock solution in 10% methanol or 10% DMSO and were tested as a suspension if not properly dissolved. Test compounds were stored at −20 °C until use, and administered in volumes of 100 µL to the plated cells. Emetine was used as the reference drug in all experiments. The highest concentration of each compound was 100 µg/mL, which was serially diluted in complete medium with 10-fold dilutions to give 6 concentrations, the lowest being 0.001 µg/L. The same dilution technique was applied to the all test samples. The highest concentration of solvent had no measurable effect on the cell viability (data not shown). Plates were developed after 44 h of exposure to the drug by the addition of a solution of MTT. After four hours, further incubation at 37 °C the supernatant was removed from the cells via suction and DMSO was added to each well to dissolve the reduced dye crystals. Plates were analysed at 540 nM wavelength using a spectrophotometer to determine the relative amount of formazan salt in each well. The amount of formazan produced in wells containing untreated cells only and growth medium only represent 100% survival and 0% survival respectively, and the amount in each treatment well was converted to a survival percentage relative to these two extremes. The IC_50_ values were obtained from full dose-response curves, using a non-linear dose-response curve fitting analysis via GraphPad Prism v.4 software.

### 3.7. Human Neuroblastoma SH-SY5Y Cell Viability Assays

#### 3.7.1. Cell Line and Culture Conditions

The human neuroblastoma cell line SH-SY5Y was generously donated by our collaborator at the Division of Molecular Biology and Human Biology, Stellenbosch University, Tygerberg, Cape Town. Cells were cultured in monolayer using Dulbecco Modified Eagles Medium (DMEM, Gibco, Life Technologies Ltd. Carlsbad, CA, USA) supplemented with 10% fetal bovine serum (FBS, Gibco, Life Technologies Ltd.) and 1% 100 U/mL penicillin and 100 μg/mL streptomycin (Lonza Group Ltd., Basel, Switzerland). Cells were grown at 37 °C, in a humidified atmosphere at 5% CO_2_. Media were replaced every two to three days and cells were sub-cultured by splitting with trypsin (Lonza Group Ltd.).

#### 3.7.2. SH-SY5Y Cytotoxicity Assays 

The standard MTT assay which measures cell metabolic activity was used to determine cytotoxicity of CM14, CM15 and CM9 as described by Mosmann [49]. Briefly, SH-SY5Y cells were plated in flat bottom 96 well plates in growth medium as stated in Section 3.7.1 at a density of 7500 cells/well. Cells were allowed to adhere to the plate surface for 24 h and used media were replaced with fresh media containing test compounds at 10 µM, 50 µM and 100 µM. Vehicle control cells were treated with DMSO (solvent for dissolving test compounds) at a concentration similar to the amount contained in the highest concentration of test compounds. After 48 h, 10 µL of MTT solution (5 mg/mL) was added to each well and incubated for 4 h and the purple formazan formed was solubilized with 100 µL DMSO and plates were read spectrophotometrically to determine absorbance at 570 nm using a BMG Labtech POLARStar Omega multimodal plate reader.

#### 3.7.3. MPP^+^-Induced Cytotoxicity in SH-SY5Y Cells

SH-SY5Y cells were seeded onto a 96-well plate and treated with 1000 μM MPP^+^ for approximately 48 h. Different concentrations (1 μM, 5 μM, and 10 μM) of test compounds were administrated two hours prior to MPP^+^ treatment. Afterwards, cell viability was measured by MTT colorimetric assay and performed as stated in Section 3.7.2.

## 4. Conclusions

As part of a collaborative project, compounds from the University of the Western Cape, School of Pharmacy drug-design group compound library were screened for antimycobacterial activity. Various coumarin derivatives, originally designed and synthesized as multifunctional neuronal enzyme inhibitors, demonstrated noteworthy antimycobacterial activity. 

The coumarin **CM9** substituted with an *N*-benzylpiperidine-containing moiety on the 7-position and a 3-nitrile substitution on the coumarin nucleus showed the best MAO-B and ChE inhibitory activities. This study identified that structural modification on position 4 and/or 7 of the coumarin scaffold can be utilized to improve selectivity towards either inhibition of neuronal enzymes or antimycobacterial effect. Large substitutions on position 4 (4-trifluoromethyl moiety) and *p*-bromo-*N*-benzylpiperazine compared to *N*-benzylpiperidine substitutions on position 7 increase activity against *M. tuberculosis* but inversely affect monoamine oxidase and cholinesterase inhibition. 

After validation of the antimycobacterial activity observed in the MTS; **CM8, CM12, CM14** (4-metyl substituted) and **CM15** (4-trifluoromethyl-substituted) were identified as the most active compounds in the series. In addition to activity in standard *M. tuberculosis* H37Rv, we also described activity of selected coumarin derivatives in fluoroquinolone resistant bacteria. Evaluations on three moxifloxacin resistant strains harboring mutations in the QRDR of DNA gyrase indicate that the compound in series 1 will likely maintain potency in fluoroquinolone resistant mycobacteria.

The MICs of the four most active compounds were evaluated using two methods i.e., *M. tuberculosis* H37RvMA pMSP12:GFP in GAST-Fe media [32], and *M. tuberculosis* H37Rv:pCHERRY3 [31] in 7H9+OADC. The MIC values in the GAST-Fe assay were consistently lower (2–3 fold lower for **CM8**, **CM12**, and **CM14** and 14 fold lower for **CM15**). Difference in inoculum size and the lack and presence of albumin in GAST-Fe and 7H9+OADC media respectively were suggested as primary contributing factors to the MIC variations.

As coumarin derivatives are known to bind extensively to blood-soluble proteins, albumin binding properties of **CM15** and **CM14** (with the highest and lowest MIC fold difference between assays respectively) were evaluated. Fluorescence quenching of BSA in response to compound binding was evaluated in the presence of various concentrations of the compounds. Analysis of fluorescence quenching of tryptophan in BSA indicate that both **CM14** and **CM15** form concentration dependant complexes with BSA. These results suggest that albumin binding plays a large role in the observed differences in activity between the GAST-Fe and 7H9+OADC assays used in this study. The more extensive decrease in fluorescence observed with **CM15** likely indicates a higher binding affinity between **CM15** and thus explains the large MIC differences observed for this compound. This report therefore highlights the importance of early consideration of plasma binding properties of coumarin derivatives as it was shown here to influence in vitro evaluations in addition to its more commonly considered effect on in vivo, and future pharmacokinetic behavior of the compounds.

**CM8**, **CM12**, **CM14**, and **CM15** showed moderate (CC_50_: 15.5–41.2 µM) cytotoxicity compared to the cytotoxic agent emetine (CC_50_ = 0.06 μM). These compounds however showed low selectivity indices when both the GFP and mCHERRY MIC_99_ assay results are compared to the CC_50_ results (Table 4). The compounds, in general, were slightly more selective towards the mycobacterial strain when using the GFP assay results, with **CM15** showing the best selectivity (SI = 3.16). The slight selectivity was however not retained when the results from the mCHERRY assay were used in the SI calculations. These poor selectivity indices may limit the further development of these coumarin derivatives. However, the structure-activity relationships identified in this paper may enable the design of coumarin structures with improved antimycobacterial activities and subsequently more favourable selectivity indices.

The viability of SHSY-5Y human neuroblastoma cells was also assessed at different concentrations of compounds **CM9**, **CM14**, or **CM15**. Treatment with the compounds at 10 µM did not significantly affect the viability of the SH-SY5Y cells (*p* > 0.05). However, at higher concentrations (50 µM and 100 µM) the viability of the cells was significantly (*p* < 0.001) affected. Neuroprotective ability of the three compounds were therefore evaluated at concentrations between 1 µM and 10 µM. **CM9**, **CM14**, and **CM15** all demonstrated significant and comparable cytoprotection towards MPP^+^ insults to the SH-SY5Y neural cells. As these compounds differ significantly in their neuronal enzyme inhibitory activity it can be concluded that enzyme inhibition is not the only factor that plays a role in cytoprotection and that other mechanisms of action are involved in the observed neuroprotection.

This article therefore describes the versatile neuronal enzyme inhibitory, neuroprotective and antimycobacterial nature of a range of coumarin derivatives. Importantly, comparison of corresponding structure-activity relationship, demonstrates that it is possible to reduce the promiscuous activity of the derivatives through careful modification on the coumarin scaffold. Furthermore the article highlights the importance of broader evaluation of pharmacological effects of coumarin derivatives over and above cytotoxicity assays to identify and afford early limitation of unwanted pharmacological effects. This report also shows that albumin binding properties of coumarin derivatives may impact on in vitro assay results and should therefore be taken into consideration even during early evaluations of coumarin-type compounds. 

## Figures and Tables

**Table 1:** Molecular structure and activity of coumarin derivatives series 1 and 2.

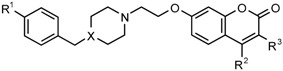					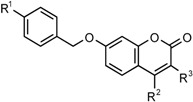		
Series 1					Series 2		
					% *M. tuberculosis* Growth, Day 5		
Number	X	R^1^	R^2^	R^3^	100 µM	50 µM	1 µM
**Series 1**							
CM12 **	N	Br	CH_3_	H	3.93	4.35	95.23
CM14 **	N	Br	CH_3_	CN	4.44	4.40	99.53
CM8 **	CH	H	CH_3_	Cl	4.62	5.69	93.59
CM15 **	N	Br	CF_3_	H	4.88	6.68	106.71
CM11 *	N	Br	H	H	4.46	7.05	95.18
CM9 *	CH	H	CH_3_	CN	6.19	7.75	92.82
CM7 *	CH	H	CH_3_	H	8.18	29.50	95.10
**CM6**	CH	H	H	H	15.43	52.71	98.19
CM13 *	N	Br	CH_3_	Cl	32.06	42.47	97.03
**Series 2**							
CM17 *		H	CH_3_	H	19.81	28.65	97.59
CM2 *		Br	CH_3_	H	33.16	40.86	89.98
CM4 *		Br	CH_3_	CN	33.52	44.60	95.27
**CM5**		Br	CF_3_	H	56.09	65.04	106.87
**CM19**		H	CH_3_	CN	55.44	66.92	98.28
**CM16**		H	H	H	50.66	72.70	97.53
**CM1**		Br	H	H	63.63	79.77	96.28
**CM18**		H	CH_3_	Cl	75.52	87.91	97.30
**CM3**		Br	CH_3_	Cl	92.86	96.11	97.82

* Compounds that underwent further analysis; ** Compounds selected for MIC determination.

**Table 2:** Minimum inhibitory concentrations determined using two methods.

Compound	GFP GAST-Fe		mCHERRY—7H9+OADC			
	MIC_90_ (µM)	MIC_99_ (µM)	MIC_90_ (µM)	St. Dev.	MIC_99_ (µM)	St. Dev.
**CM8**	19.6	25.2	40.55	1.16	44.15	1.37
**CM12**	14	29.7	40.89	0.06	50.40	8.96
**CM14**	16.7	28	45.22	3.04	54.46	9.58
**CM15**	3.2	8.31	43.78	1.46	57.17	10.05

Abbreviations: St. Dev., standard deviation; MIC90, minimum inhibitory concentration required to inihibit 90 % of the organism; MIC_99_, minimum inhibitory concentration required to inhibit 99% of the organism; GFP, green fluorescent protein; GAST-Fe, glycerol-alanine-salts with 0.05% Tween 80 and iron; 7H9+OADC, Middlebrook 7H9 broth, enriched with 10% oleic albumin dextrose catalase (OADC), 0.2% glycerol and 0.05% Tween 80.

**Table 3:** Excitation and emission maxima of **CM14** and **CM15**, and the quenching constant of BSA by **CM14** and **CM15**.

Compound	T (°C)	*λ*_Ex_ (nm)	*λ*_Em_ (nm)	k_sv_ (mol L^−1^)	k_q_ (mol L^−1^ S^−1^)	R^2^
**CM14**	22	350 ^a^ 350 ^b^	395 ^a^ 400 ^b^	3.80 × 10^5^	2.63 × 10^14^	0.9780
**CM15**	22	335 ^a^ 335 ^b^	415 ^a^ 415 ^b^	4.88 × 10^5^	2.00 × 10^14^	0.9927

^a^ Measured in Tris-HCl buffer at 1 × 10^−6^ mol L^−1^. ^b^ Measured in BSA (1 × 10^−5^ mol L^−1^) at 1 × 10^−6^ mol L^−^^1^. Abbreviations: *λ*_Ex_: excitation; *λ*_Em_: emission; k_sv_: dynamic quenching constant; k_q_: quenching rate constant; R^2^: coefficient of determination.

**Table 4:** 50% cell viability (CC_50_) and selectivity indices on Chinese hamster ovary (CHO) cells, cell viability on SH-SY5Y cells and 50% enzyme inhibition (IC_50_) of selected test compounds.

CM	CC_50_ µM CHO	SI GFP ^a^	SI mCHERRY ^b^	CC 50% SH-SY5Y ^c^	MAO-A IC_50_ µM ^d^	MAO-B IC_50_ µM ^d^	AChE IC_50_ µM ^d^	BuChE IC_50_ µM ^d^
**CM8**	33.0	1.31	0.75	ND	n.a.	0.29	31.30	1.27
**CM9**	ND	ND	ND	10–50	n.a.	0.30	9.10	5.90
**CM12**	15.5	0.52	0.31	ND	n.a.	3.60	12.8	9.70
**CM14**	41.2	1.47	0.76	50–100	n.a.	1.41	38.5	13.3
**CM15**	26.3	3.16	0.46	10–50	n.a.	5.64	>100	>100
**Emetine**	0.06	ND	ND	ND	ND	ND	ND	ND

^a^ Selectivity index (SI) = CC_50_ CHO/MIC_99_ GFP-GAST-Fe. ^b^ Selectivity index (SI) = CC_50_ CHO/MIC_99_ mCHERRY-7H9+OADC. ^c^ Inhibition range (uM) where 50% cell toxicity will be observed. ^d^ Data taken from Joubert et al., 2017 [23]. ND = not determined. n.a. = no activity.
